# Exosomal hsa-miR-21-5p is a biomarker for breast cancer diagnosis

**DOI:** 10.7717/peerj.12147

**Published:** 2021-09-17

**Authors:** Min Liu, Fei Mo, Xiaohan Song, Yun He, Yan Yuan, Jiaoyan Yan, Ye Yang, Jian Huang, Shu Zhang

**Affiliations:** 1 Department of Laboratory Medicine, Sichuan Maternal and Child Health Hospital, Chengdu, Sichuan Province, China; 2 Department of Clinical Laboratory, Affiliated Hospital of Guizhou Medical UniversityGuiyang, Guizhou Province, China; 3 Department of Basic Clinical Laboratory Medicine, School of Clinical Laboratory Science, Guizhou Medical University, Guiyang, Guizhou Province, China

**Keywords:** Breast cancer, Diagnosis, Bioinformatics, Biomarker, microRNA, Exosome

## Abstract

**Purpose:**

Breast cancer (BC) is characterized by concealed onset, delayed diagnosis, and high fatality rates making it particularly dangerous to patients’ health. The purpose of this study was to use comprehensive bioinformatics analysis and experimental verification to find a new biomarker for BC diagnosis.

**Methods:**

We comprehensively analyzed microRNA (miRNA) and mRNA expression profiles from the Gene Expression Omnibus (GEO) and screened out differentially-expressed (DE) miRNAs and mRNAs. We used the miRNet website to predict potential DE-miRNA target genes. Using the Database for Annotation, Visualization and Integrated Discovery (DAVID), we performed Gene Ontology (GO) and the Kyoto Encyclopedia of Genes and Genomes (KEGG) analyses on overlapping potential target genes and DE-mRNAs. The protein-protein interaction (PPI) network was then established. The miRNA-mRNA regulatory network was constructed using Cytoscape and the analysis results were visualized. We verified the expression of the most up-regulated DE-miRNA using reverse transcription and a quantitative polymerase chain reaction in BC tissue. The diagnostic value of the most up-regulated DE-miRNA was further explored across three levels: plasma-derived exosomes, cells, and cell exosomes.

**Results:**

Our comprehensive bioinformatics analysis and experimental results showed that hsa-miR-21-5p was significantly up-regulated in BC tissue, cells, and exosomes. Our results also revealed that tumor-derived hsa-miR-21-5p could be packaged in exosomes and released into peripheral blood. Additionally, when evaluating the diagnostic value of plasma exosomal hsa-miR-21-5p, we found that it was significantly up-regulated in BC patients. Receiver operating characteristic (ROC) analysis also confirmed that hsa-miR-21-5p could effectively distinguish healthy people from BC patients. The sensitivity and specificity were 86.7% and 93.3%, respectively.

**Conclusion:**

This study’s results showed that plasma exosomal hsa-miR-21-5p could be used as a biomarker for BC diagnosis.

## Introduction

Breast cancer (BC) is a malignant tumor that originates from the glandular epithelium of the breast. Due to the lack of typical and specific clinical symptoms and signs in the early stage, most patients present with symptoms and are diagnosed in middle and late stages, making BC particularly dangerous. Although BC is the leading cause of cancer-related death in women (Bray et al., 2018), the current detection methods used to clinically diagnose BC are inadequate to a certain extent. Specifically, pathologic examinations are invasive and imaging examinations are not accurate. The sensitivity and/or specificity of existing biomarkers requires further analysis, which limits their application in BC diagnosis (Bevers et al., 2018; Holloway et al., 2010; Lian et al., 2019). Therefore, new biomarkers are needed to promptly detect and diagnose BC in order to improve the rate of survival of BC patients (De Santis et al., 2016).

MicroRNAs (miRNAs) are non-coding small RNAs in eukaryotes that are approximately 21 to 23 nucleotides long. MiRNAs have been found to be heavily dysregulated in many types of malignant tumors and to participate in a series of important biological activities, such as tumor cell proliferation (Chen et al., 2020; Roy et al., 2018; Wei & Gao, 2019), migration (Chen et al., 2020; Wei & Gao, 2019; Xu, Yu & Liu, 2020; Yu et al., 2019), and apoptosis (Chen et al., 2019; Kasinski & Slack, 2011). It has been reported that miR-1246 targets CaV1 regulation and acts on the PDGFβ receptor in ovarian cancer cells, thereby inhibiting cell proliferation (Kanlikilicer et al., 2018). Hong et al. (2019) found that miR-204-5p directly regulates PIK3Cβ expression and the downstream PI3K/Akt signal pathway in BC, hence affecting BC growth and metastasis. Therefore, understanding tumor-related miRNAs might help reveal the molecular mechanisms underlying tumor development and formation, and provide new insights for clinical tumor diagnosis and treatment.

Exosomes are lipid bimolecular vesicles with a diameter of 30–150 nm (Deng & Miller, 2019). Exosomes exist in plasma, urine, saliva, and cell supernatant culture medium, and are released into the extracellular space by a variety of cells during exocytosis (Hesari et al., 2018). They contain receptors on their lipid bilayer membrane and carry proteins, lipids, mRNAs, miRNAs, and long non-coding RNAs derived from the original inside cells to protect them from degradation (De Veirman et al., 2016; Di Pace et al., 2020; Wang, Zheng & Zhao, 2016). Recently, a growing body of evidence shows that exosomal nucleic acids can act as novel biomarkers to diagnosis many types of diseases (Deng, Magee & Zhang, 2017). Exosome-derived miR-192, miR-122, and miR-30a showed excellent diagnostic value in identifying alcoholic hepatitis (AH), confirming that they are promising biomarkers for AH diagnosis (Momen-Heravi et al., 2015). Chen et al. (2015) found that plasma exosomal miR-214 is a potential biomarker for liver fibrosis.

**Figure 1:**
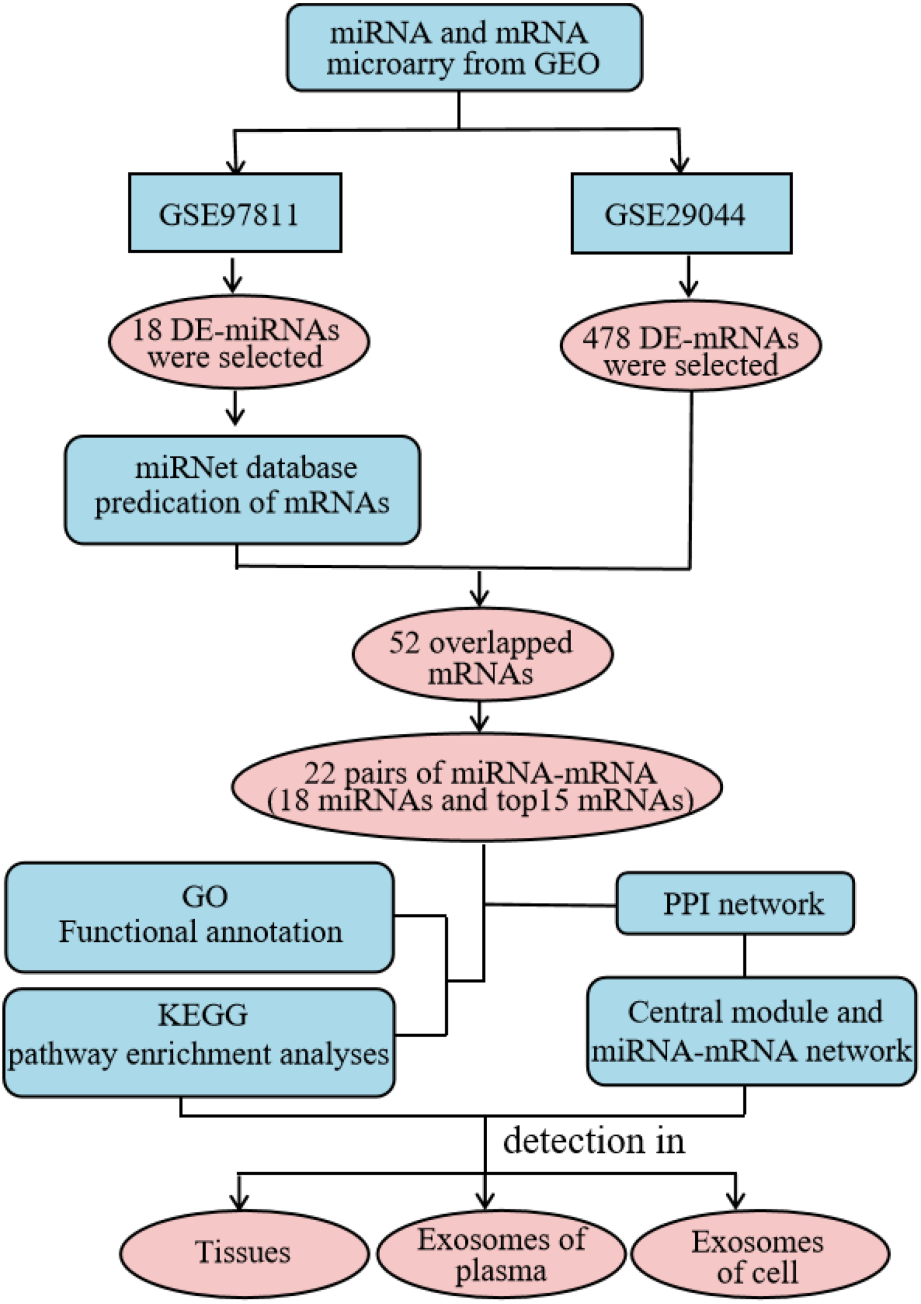
Detailed process of the proposed method. PPI, protein–protein interaction.

High throughput microarray technology for expression profiling has become widely-used in the study of cancer genes and the identification of new biomarkers (Huang & Gao, 2018). In this study, we comprehensively analyzed the expression profile of the Gene Expression Omnibus (GEO) database to obtain differentially-expressed (DE) miRNAs and mRNAs. We used the miRNet website to predict potential DE-miRNA target genes. Gene Ontology (GO) annotation and Kyoto Encyclopedia of Genes and Genomes (KEGG) pathway analysis were performed on the intersection of potential target genes and DE-mRNAs using the the Database for Annotation, Visualization and Integrated Discovery (DAVID) database, and the protein–protein interaction (PPI) network was constructed using the STRING database. Additionally, the miRNA-mRNA regulatory network was constructed using Cytoscape and the results were visualized. Using our analysis, we explored the expression and diagnostic value of significantly different miRNAs across three specimen types (tissue, plasma, and cellular exosomes). This provided a basis for studying the molecular mechanisms underlying BC progression and identifying reliable biomarkers for diagnosis (Fig. 1).

## Materials & Methods

### Microarray dataset acquisition

The miRNA expression profile of GSE97811 and the mRNA expression profile of GSE29044 were downloaded from the GEO database (http://www.ncbi.nlm.nih.gov/geo/). GSE97811 was detected using the GPL21263 3D-Gene Human miRNA V21_1.0.0 platform, which included 16 normal controls and 45 BC tissues. GSE29044 was detected using the GPL570 [HG- U133_Plus_2] Affymetrix Human Genome U133Plus 2.0 Array platform, which included 36 normal controls and 73 BC tissues.

### Identifying DE-miRNAs and DE-mRNAs

The GEO2R online tool (https://www.ncbi.nlm.nih.gov/geo/info/geo2r.html) was used to analyze and filter miRNAs (DE-miRNAs), which were significantly different in GSE97811, and mRNAs (DE-mRNAs), which were significantly different in GSE29044. —log2FC— ≥2 and *P* <0.05 were considered the cutoff values.

### Predicting DE-miRNAs targeting mRNAs and acquisition of overlapping mRNAs

We used miRNet (https://www.mirnet.ca/) to predict the target mRNAs of the DE-miRNAs (Fan et al., 2016). The predicted target mRNAs were matched with the DE-mRNAs obtained by microarray analysis, and the overlapping mRNAs were obtained using the Venn online tool (http://bioinformatics.psb.ugent.be/webtools/Venn/).

### Functional enrichment analysis

GO and KEGG pathway analyses were performed using the DAVID Bioinformatics Resource (version 6.8, https://david.ncifcrf.gov/), a database for annotation, visualization, and integrated discovery (Huang, Sherman & Lempicki, 2009a; Huang, Sherman & Lempicki, 2009b). The DAVID Bioinformatics Resource was helpful for understanding the biological functions and possible pathways of overlapping target genes. The results were visualized using the R software package (ggplot2 and Cairo). *P* < 0.05 was considered statistically significant.

### Constructing the PPI and miRNA-mRNA network

We used STRING (version 11.0, https://string-db.org/cgi/network.pl) to draw the PPI network, and we set the cut-off criterion to a confidence score >0.4 (Szklarczyk et al., 2019). We used the Cytoscape ‘cytoHubba’ plug-in (v3.6.0, http://cytoscape.org) to screen the hub genes by degree value, then constructed and identified the top 10 miRNA-mRNA networks using the hub genes and DE-miRNAs.

### Expressing potential target genes and drawing a Kaplan-Meier curve

We used UALCAN (http://ualcan.path.uab.edu/index.html) and the human protein atlas (HPA, https://www.proteinatlas.org/) to search for the expression of potential miRNA target genes with the most significant differences (Chandrashekar et al., 2017; Uhlen et al., 2017). The Kaplan Meier Plotter online tool (http://kmplot.com/analysis/) was used to analyze data from 1,078 BC samples taken from the Cancer Genome Atlas database (Nagy et al., 2018). Using the tool we explored the relationship between the target genes and the BC patients’ prognosis.

### Sample collection

This study was approved by the Ethics Committee of the Affiliated Hospital of Guizhou Medical University and carried out in accordance with the ethical standards of the Declaration of Helsinki (2019032K and 2019033K). All patients signed informed consent. The samples included surgical tissue and peripheral blood samples from 30 patients with BC (all confirmed by pathologic evaluation, all females, age 36–69 years, with an average age of 55.5 years). The surgical tissues included BC and adjacent tissue (3 ∼5 cm from the tumor margin). Simultaneously, peripheral blood samples were collected from 30 healthy patients (all females, age 25–65 years, with an average of 45.5 years). Six peripheral blood samples were each obtained from patients with liver cancer (three males and three females, age 45–70 years, with an average age of 62.5 years), lung cancer (three males and three females, age 47–75 years, with an average age of 68.3 years), cervical cancer (all females, age 47–60 years, with an average age of 53.2 years), and ovarian cancer (all females, age 47–58 years, with an average age of 51.2 years).

### Cell culture

Human non-tumorigenic epithelial cell line MCF 10A and BC cells MDA-MB-23, MCF7, and BT474 were purchased from the American Type Culture Collection (ATCC). MCF10A cells were cultured in an MEGM kit (CC3150; Lonza) with 100 ng/mL cholera toxin (C8052; Sigma). MDA-MB-231 and MCF7 cells were cultured in DMEM medium (Gibco, C11995500BT) and BT474 was cultured in RPMI-1640 medium (C11875500BT; Gibco). We added 10% exosome- free fetal bovine plasma and antibiotics (100 U/mL of penicillin and 100 U/mL of streptomycin) to the medium, and cultured it at 37 °C in a 5% CO_2_ incubator.

### Isolating and identifying exosomes

Peripheral blood was centrifuged at 4  ° C for 1200 ×g for 10 min to obtain plasma. Plasma-derived exosomes were isolated through differential ultracentrifugation (Théry et al., 2006). The exosome morphology was analyzed by transmission electron microscopy (TEM). Ten minutes after 10 µL of the exosomes were pipetted onto a grid coated with formvar and carbon at room temperature, excess fluid was removed, and the sample was negatively stained with 3% phosphotungstic acid (pH 6.8) for 5 min. Finally, the samples were analyzed by TEM. The exosome concentration and size range were identified using a NanoSight NS300 system (NanoSight, Salisbury, United Kingdom) supplied with a fast video capture and Nanoparticle Tracking Analysis (NTA) software. Before performing the experiments, the instrument was calibrated with 100 nm polystyrene beads (Thermo Scientific, Waltham, MA, USA). The samples were captured for 60 s at room temperature. NTA software processed the video captures and measured the particle concentration (particles/ml) and size distribution (in nanometer). Each specimen was measured three times.

### Western blotting

The total exosomal proteins were extracted and then subjected to SDS-PAGE electrophoresis to transfer the protein to the PVDF membrane. The 5% skimmed milk was sealed at room temperature for 2 h, washed three times with TBST, incubated overnight at 4 °C with TSG101 (ab125011; Abcam) and Calnexin (ab133615; Abcam) antibody, and incubated for 1 h in HRP-labeled secondary antibody (SA00001-2; Proteintech) at room temperature. After washing three times, the exosome protein marker expression was detected using ECL luminescence and Calnexin as the negative control.

### Reverse transcription and quantitative polymerase chain reaction (RT-qPCR) analysis

Total RNA was extracted from tissues, cells, and exosomes using the TRIzol™ Reagent (Invitrogen, 11596026) according to the manufacturer’s instructions. The primers used in the study (Table S1) were synthesized by Sangon Biotech (Shanghai, China). Reverse transcription was performed in accordance with the PrimeScript™ RT Reagent Kit (RR037A; TaKaRa) manufacturer’s instructions. We performed DE-miRNA RT-qPCR using TB Green©PreMix Ex Taq™ II (RR820A; TaKaRa) with corresponding forward and reverse primers. U6 snRNA was used for normalization, and DE-miRNA relative expression was calculated using the 2^−ΔΔCt^ method.

### ROC curve analysis of the most significant difference in miRNA performance in BC diagnosis

RT-qPCR was used to determine the expression of DE-miRNAs in BC and adjacent tissue, and the miRNAs with the most significant differences were selected. We used a receiver operating characteristic (ROC) curve to evaluate the miRNA values as a diagnostic marker for BC. The Youden index was used to determine the cut-off value of the most significant difference in the relative expression of DE-miRNA. At the same time, we randomly selected samples from six healthy people, six BC patients, six liver cancer patients, six lung cancer patients, six cervical cancer patients, and six ovarian cancer patients to detect the miRNA expression with the most significant difference in plasma-derived exosomes.

### Statistical analysis

Statistical analysis was performed using SPSS24.0 software. A paired sample t test was used to compare hsa-miR-21-5p expression in BC and adjacent tissue, and in plasma-derived exosomes of BC patients before and after surgery. The Pearson correlation was used to analyze the correlation between hsa-miR-21-5p in plasma-derived exosomes and cancer tissues. *P* < 0.05 was considered statistically significant.

## Results

### Identifying DE-miRNAs and DE-mRNAs

**Figure 2:**
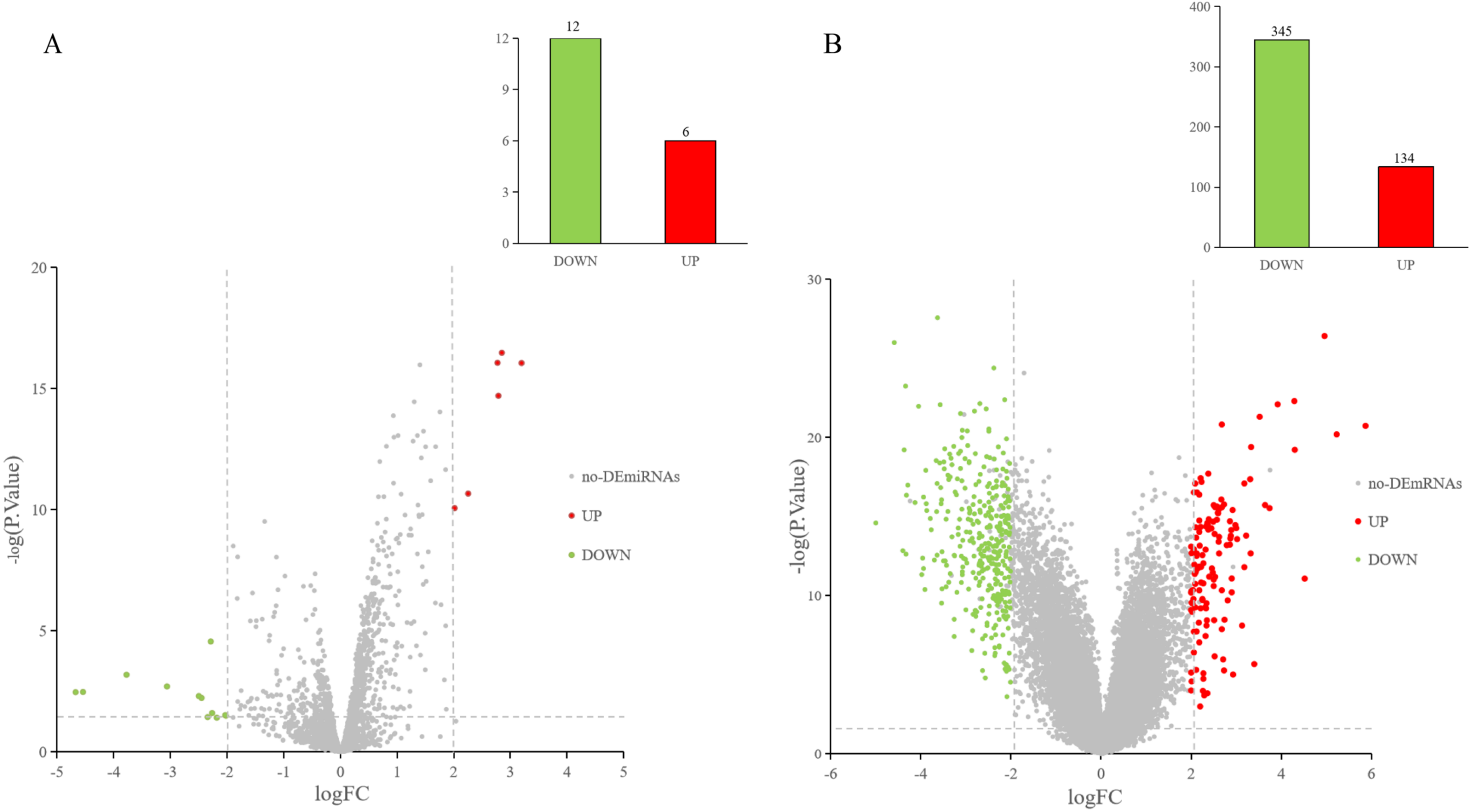
DE-miRNAs and DE-mRNAs were identified from GSE97811 and GSE29044, respectively. Red represents up-regulation; green represents down-regulation (A) Volcanic map of miRNAs in GSE97811. (B) Volcanic map of mRNAs in GSE29044.

The GEO2R online tool was used to analyze the GEO datasets. Of the 18 DE-miRNAs screened from the miRNA expression profile dataset GSE97811, six miRNAs were up-regulated and 12 miRNAs were down-regulated (Fig. 2A). After analyzing the mRNA expression dataset GSE29044, we screened 479 DE-mRNAs: 134 mRNAs were up-regulated and 345 mRNAs were down-regulated (Fig. 2B).

### Predicting target genes and overlapping mRNA acquisition

**Figure 3:**
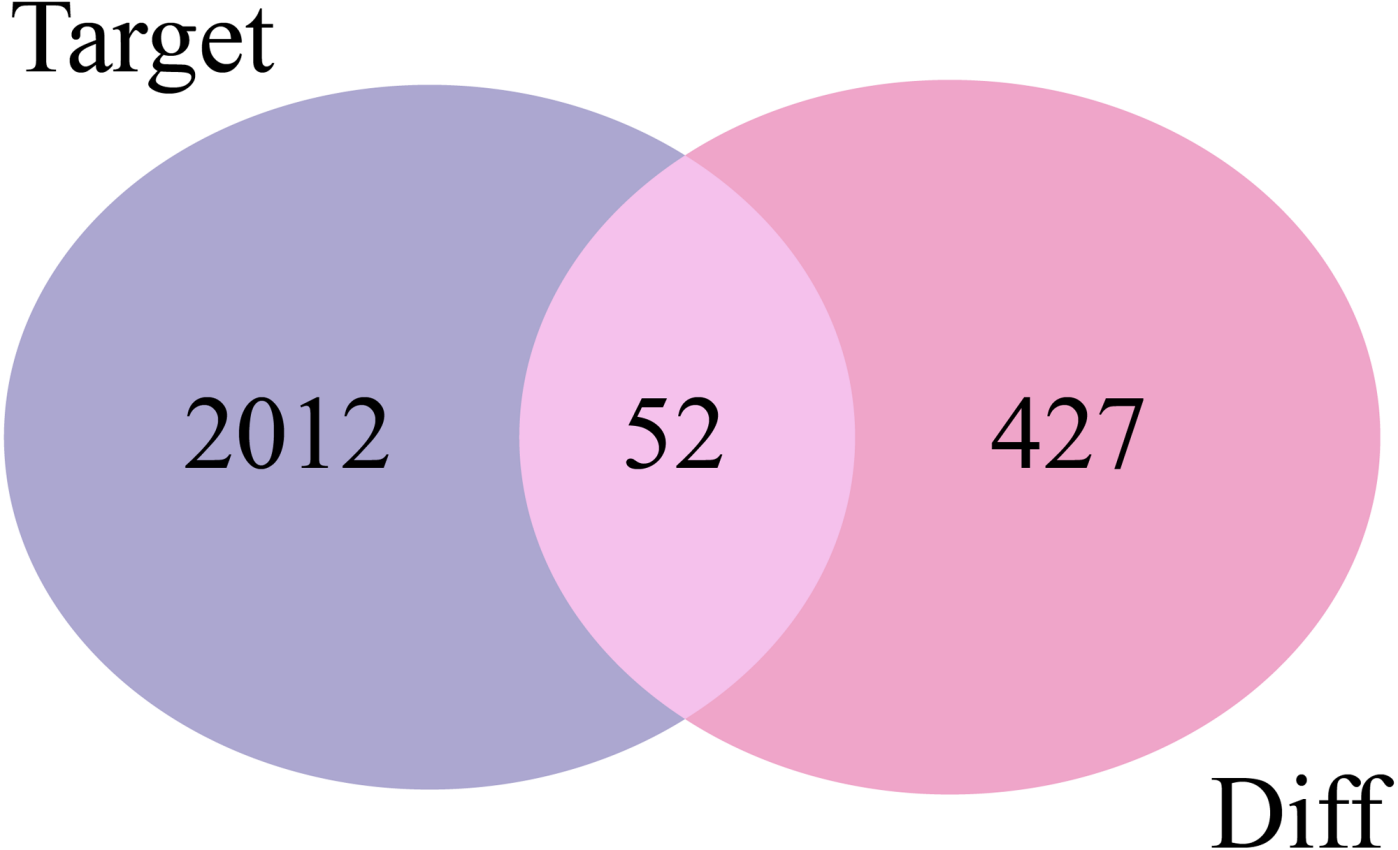
Venn diagrams of target mRNAs and DE-mRNAs. Target, predicted target mRNAs; Diff, DE-mRNAs obtained by GSE29044.

The 2,064 target mRNAs of the DE-miRNAs predicted by miRNet (Table 1) were matched with the 479 DE-mRNAs obtained by GSE29044, and 52 overlapping mRNAs were identified for further analysis (Fig. 3).

### Functional enrichment analysis of overlapping mRNAs

**Figure 4:**
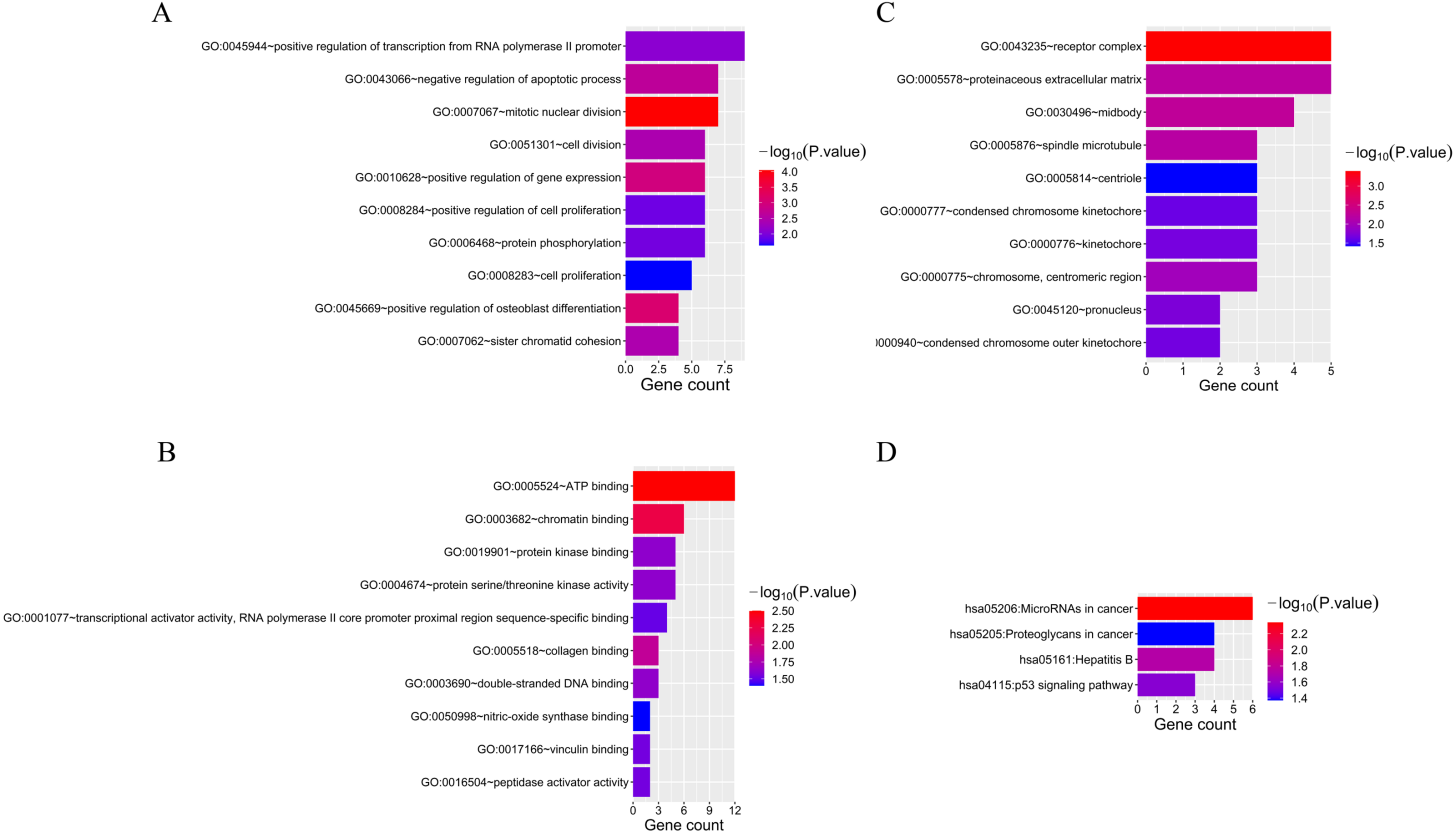
The results of GO and KEGG analyses of 52 overlapping mRNAs. (A) The top 10 GO terms in the BP results of 52 overlapping mRNAs. (B) The top 10 GO terms in the MF results of 52 overlapping mRNAs. (C) The top 10 GO terms in the CC results of 52 overlapping mRNAs. (D) The results of KEGG pathway enrichment analysis of 52 overlapping mRNAs.

Fifty-two overlapping mRNAs were analyzed. Biological process (BP) included the positive regulation of transcription from RNA polymerase II promoter, cell division, and cell proliferation (Fig. 4A). Molecular function (MF) mainly included ATP and chromatin binding (Fig. 4B). The cellular components (CC) were mainly receptor complexes and proteinaceous extracellular matrix (Fig. 4C). Additionally, the KEGG pathway was mainly enriched in cancer-related pathways (Fig. 4D), such as miRNAs in cancer (hsa05206), proteoglycans in cancer (hsa05205), and the p53 signaling pathway (hsa04115).

**Table 1:** Eighteen differentially expressed miRNAs and predicted number of target genes.

**miRNAs**	**Number of target genes**	**Type**
hsa-miR-22-3p	163	Up
hsa-miR-429	151	Up
hsa-miR-96-5p	201	Up
hsa-miR-21-5p	612	Up
hsa-miR-425-5p	144	Up
hsa-miR-182-5p	179	Up
hsa-miR-4256	35	Down
hsa-miR-5481	172	Down
hsa-miR-3201	40	Down
hsa-miR-380-3p	77	Down
hsa-miR-5007-5p	112	Down
hsa-miR-4772-5p	58	Down
hsa-miR-136-3p	48	Down
hsa-miR-219a-5p	46	Down
hsa-miR-208b-5p	111	Down
hsa-miR-4650-5p	86	Down
hsa-miR-4645-3p	71	Down
hsa-miR-205-5p	182	Down

### Constructing the PPI and miRNA-mRNA networks

**Figure 5:**
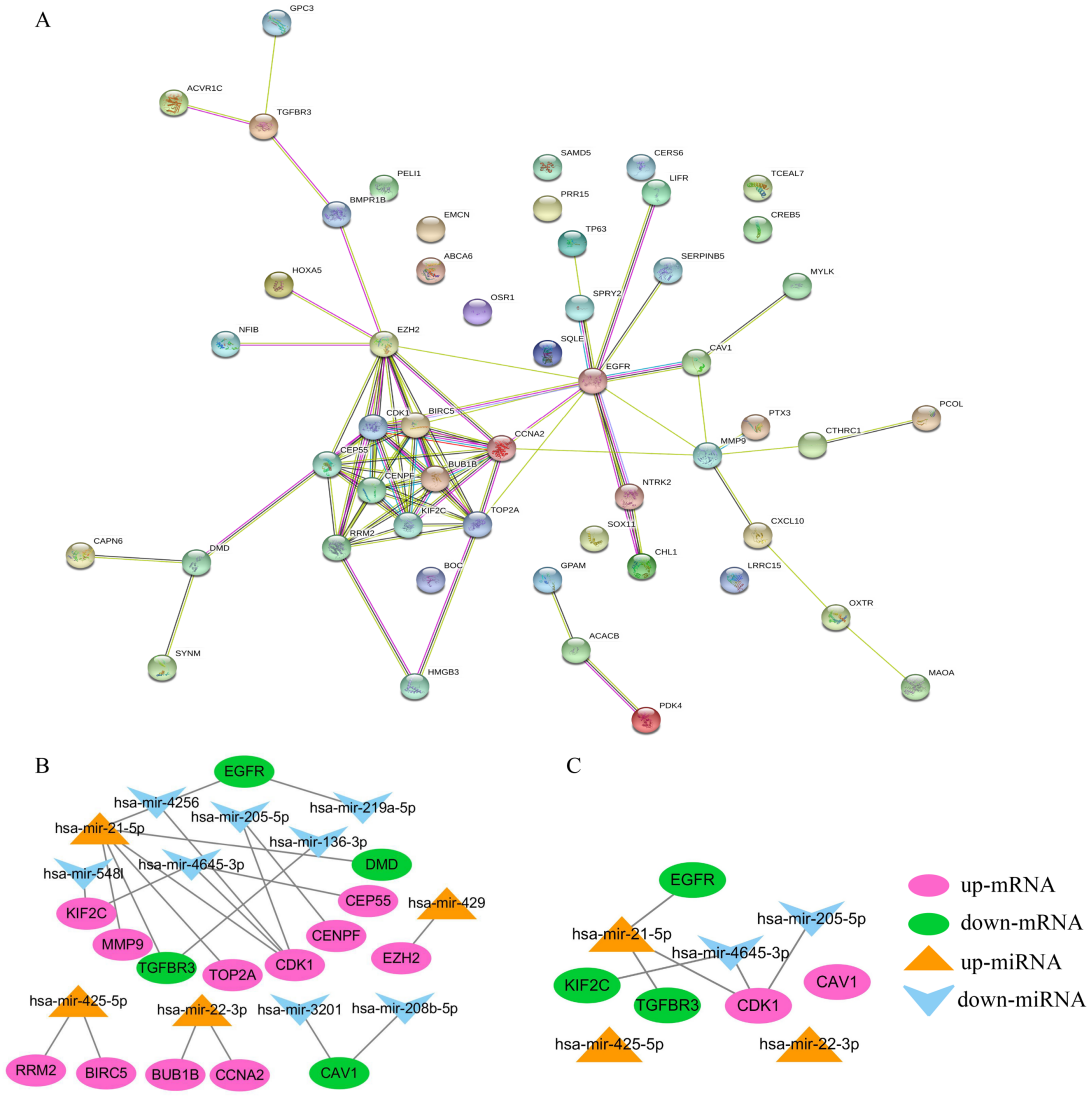
Construction of PPI and miRNA-mRNA networks. PPI: protein–protein interaction. (A) PPI network of 52 overlapping mRNAs. (B) The miRNA-mRNA network constructed by 15 hub genes and DE-miRNAs. (C) The identified degree ranks the top 10 miRNA-mRNA networks.

STRING was used to construct the PPI network of 52 overlapping mRNAs, which included 52 nodes and 81 edges (Fig. 5A). The Cytoscape cytoHubba plug-in was used to identify the top 15 hub genes in this network (Table 2). These 15 hub genes and DE-miRNAs were used to draw the miRNA-mRNA network, which included has-miR-4645-3p, hsa-miR-205-5p, hsa-miR-21-5p, hsa-miR-22-3p, and hsa-miR-425-5p (Fig. 5B), in which *EGFR*, transforming growth factor beta receptor III (*TGFβR3*), *CDK1*, and *KIF2C* were potential target genes of hsa-miR-21-5p, hsa- miR-205-5p, and has-miR-4645-3p, respectively (Fig. 5C). No potential target genes of hsa-miR- 22-3p or hsa-miR-425-5p were found.

**Table 2:** The top 15 mRNAs in the PPI network and their degree.

**mRNA**	**Degree**	**Type**
EZH2	13	Up
TOP2A	11	Up
CCNA2	11	Up
CDK1	11	Up
RRM2	10	Up
BIRC5	10	Up
KIF2C	9	Up
CENPF	9	Up
CEP55	9	Up
BUB1B	9	Up
MMP9	6	Up
EGFR	13	Down
CAV1	3	Down
TGFBR3	3	Down
DMD	3	Down

### hsa-miR-21-5p expression was significantly up-regulated in BC tissue and cells

**Figure 6:**
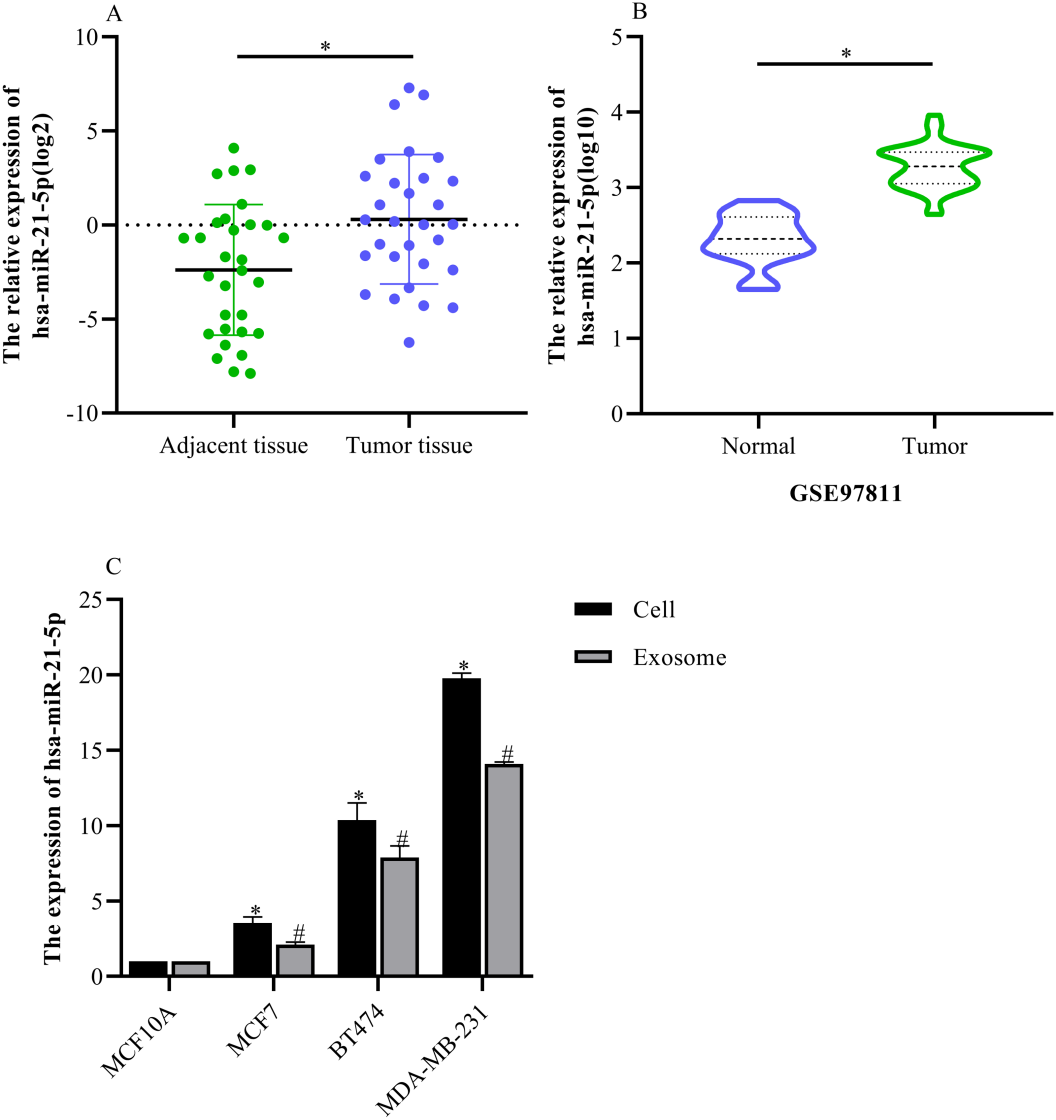
The expression of hsa-miR-21-5p was significantly up-regulated in breast cancer tissues and breast cancer cells. (A) The expression of hsa-miR-21-5p in 30 pairs of breast cancer tissues and corresponding adjacent tissues. (B) The expression of hsa-miR-21-5p in GSE97811. (C) The expression of hsa-miR-21-5p in breast cancer cell lines. ^∗^*P* < 0.05 *vs.* a MCF10A cell group; ^#^*P* < 0.05 *vs.* a MCF10A exosome group.

Based on our analysis, we selected hsa-miR-21-5p for follow-up study because it had the greatest up-regulated expression. The RT-qPCR results showed that hsa-miR-21-5p expression was up- regulated in BC tissue when compared with adjacent tissue (Fig. 6A), which was consistent with our results from the GEO database (Fig. 6B). Additionally, our results showed that hsa-miR-21- 5p expression in BC cells and the derived exosomes was up-regulated, especially in MDA-MB- 231 cells (Fig. 6C).

### Exosome identification

**Figure 7:**
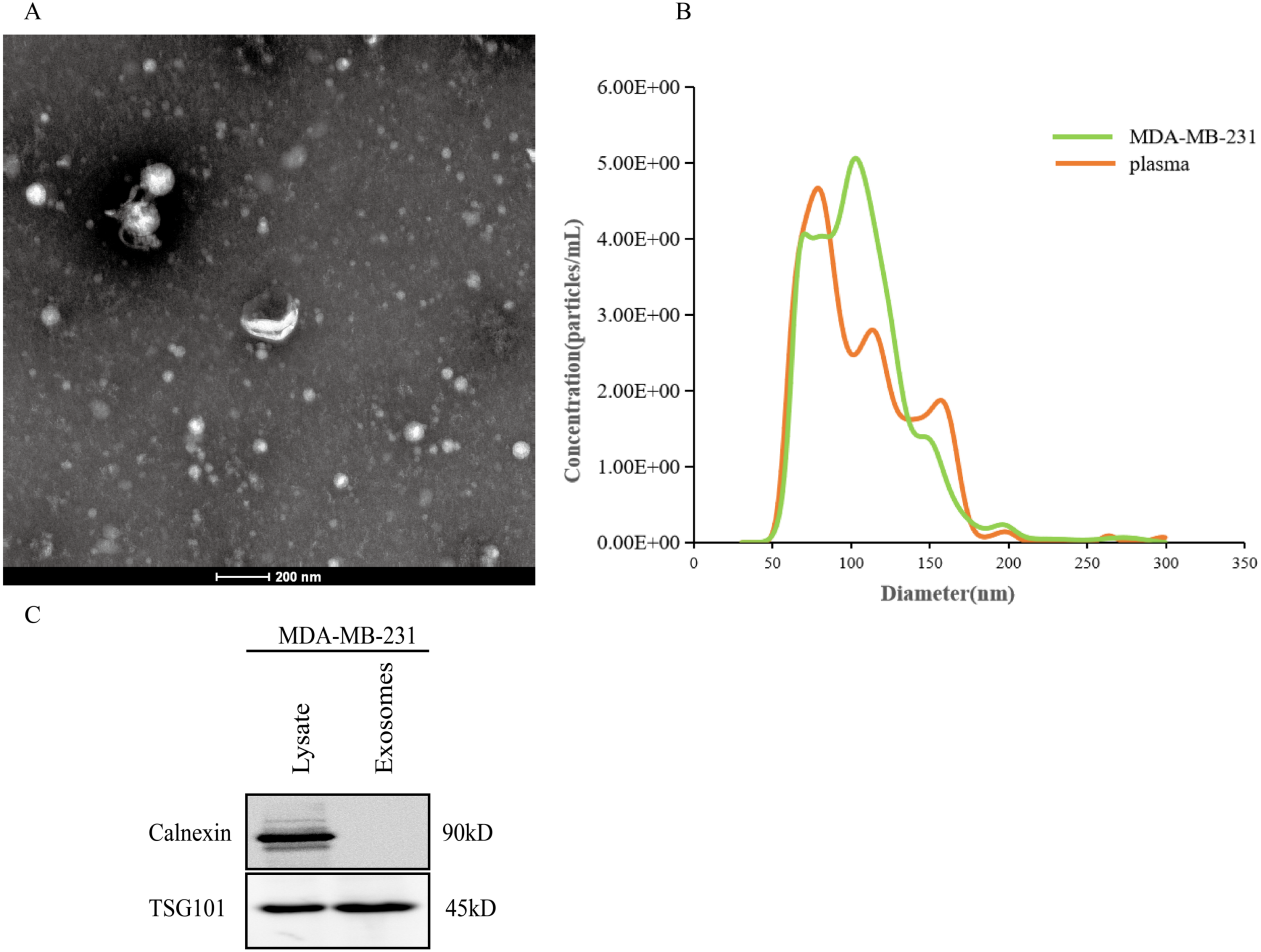
Extraction and identification of exosomes. (A) TEM characteristics of exosomes. (B) The distribution of exosome particles. (C) The results of western blotting characterization of exosomes.

After the exosomes were isolated from plasma, we used TEM to examine the exosomes and found that they were typically dish-shaped and contained low electron density substances (Fig. 7A). NTA showed that the exosome particle size was between 100 and 300 nm and was relatively uniform, and the diameter was approximately 128 nm (Fig. 7B). Western blotting detected exosome protein marker TSG101. Calnexin was not expressed (Fig. 7C). The results indicated that the exosomes were successfully extracted.

### Elevated plasma hsa-miR-21-5p was tumor-derived and packaged into exosomes

**Figure 8:**
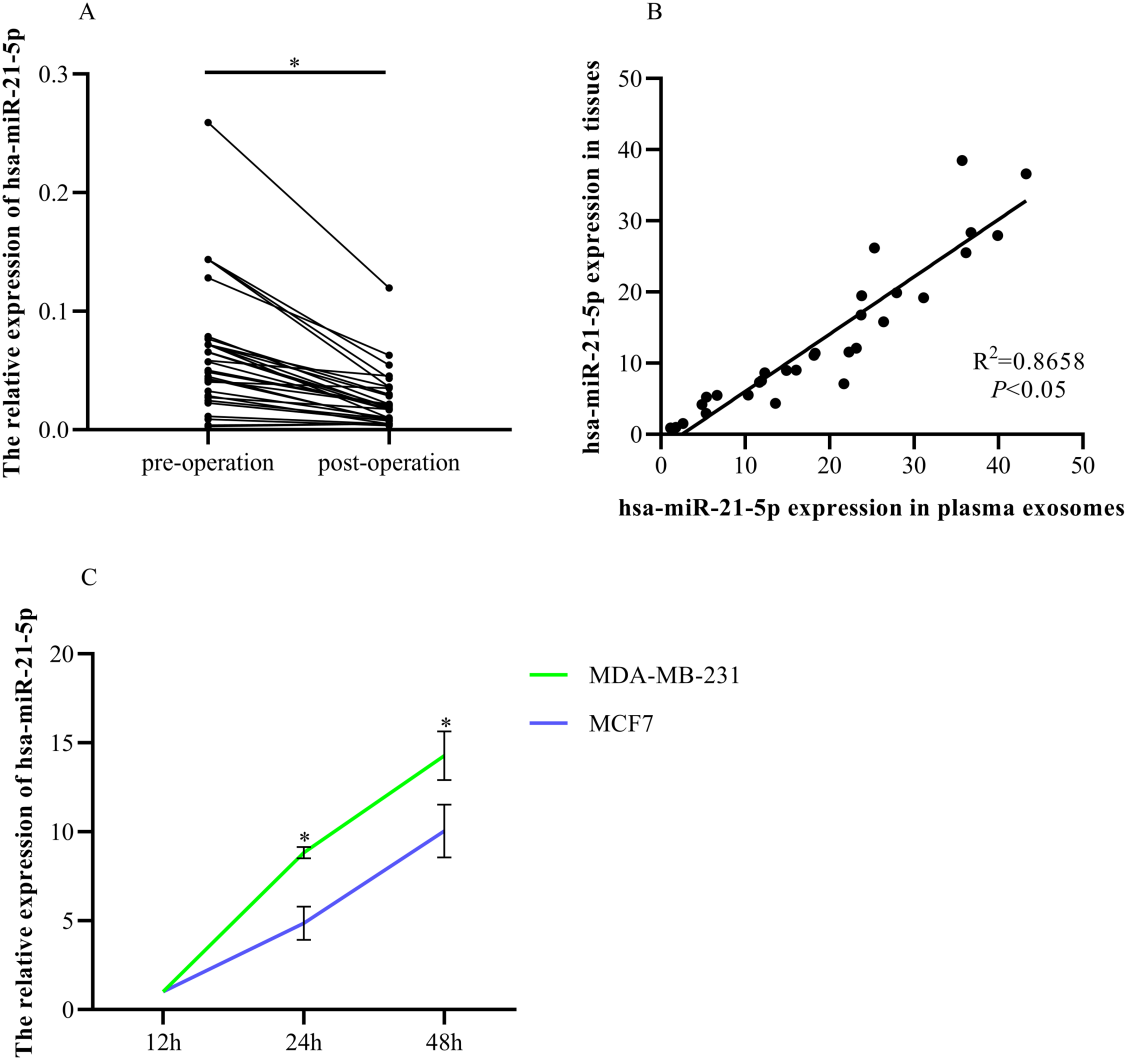
Elevated plasma hsa-miR-21-5p was tumor-derived after packaging into exosomes. (A) Expression of hsa-miR-21-5p in plasma exosomes of 30 patients with breast cancer before and after surgery. (B) The expression of hsa-miR-21-5p in plasma exosomes of patients with breast cancer was positively correlated with it in breast cancer tissues. (C) The expression of hsa-miR-21-5p in MDA-MB-231 and MCF7 exosomes varied with the duration of culture, ^∗^*P* < 0.05.

RT-qPCR was used to determine hsa-miR-21-5p expression in the plasma-derived exosomes of BC patients before and after tumor resection. The expression was significantly down-regulated after tumor resection (Fig. 8A). Additionally, we found that plasma exosomal hsa-miR-21-5p expression in BC patients was positively correlated with has-miR-21-5p expression in BC tissue (Fig. 8B). We then cultured MDA-MB-231 and MCF7 cells, collected exosomes in the culture medium at different time points, and noted that exosome hsa-miR-21-5p expression in the two cells’ culture media increased over time (Fig. 8C).

### Exosomal hsa-miR-21-5p was significantly up-regulated in BC patient plasma and had remarkable diagnostic value

**Figure 9:**
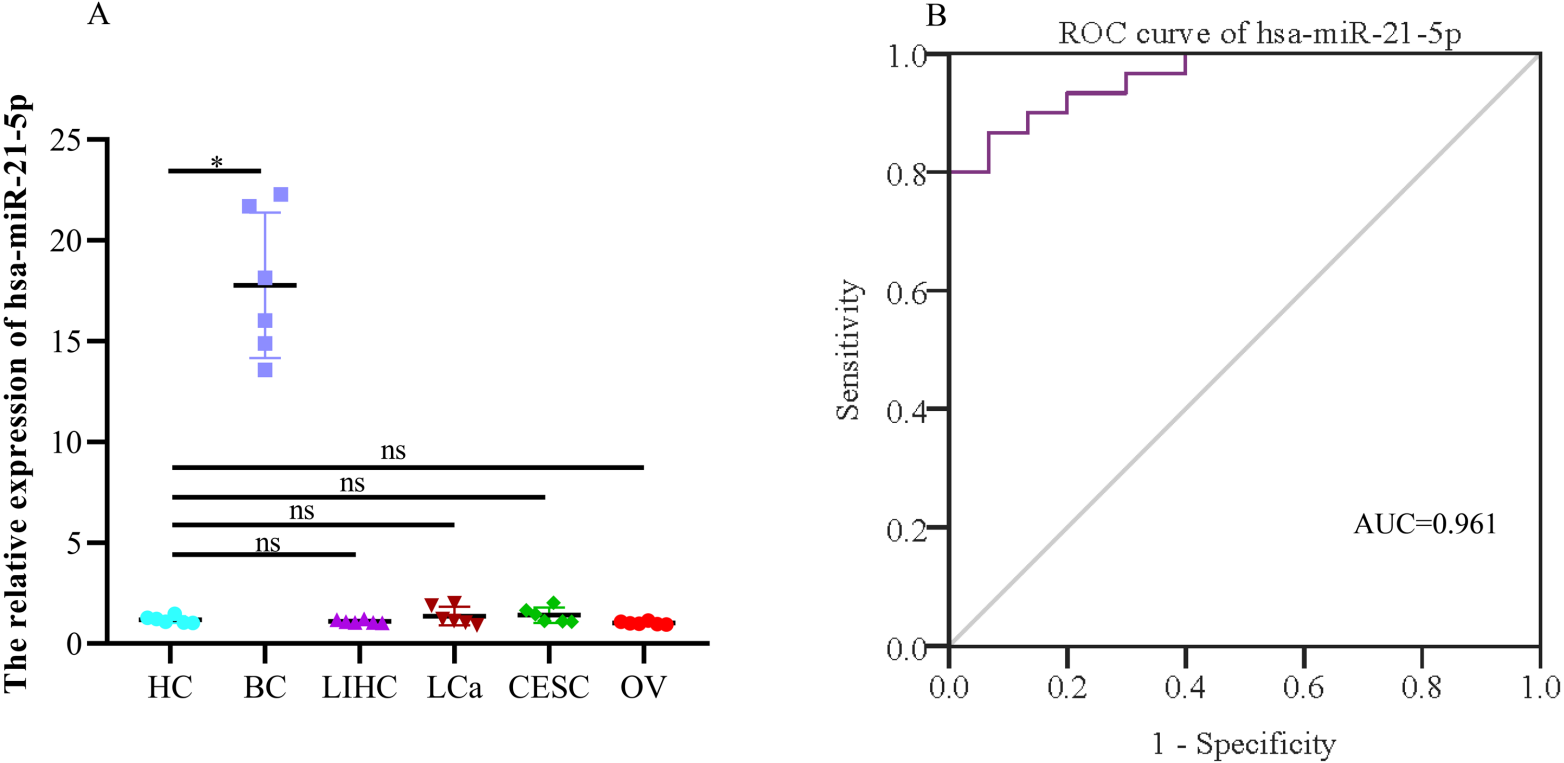
The expression of the exosome hsa-miR-21-5p of plasma and its diagnostic value. (A) hsa-miR-21-5p in plasma exosomes of other cancer patients, HC, healthy control;BC, breast cancer; LIHC, liver hepatocellular carcinoma; LCa, lung cancer; CESC, cervical squamous cell carcinoma and endocervical adenocarcinoma; OV, ovarian serous cystadenocarcinoma. (B) An ROC curve was used to analyze the sensitivity and specificity of hsa-miR-21-5p in the diagnosis of breast cancer. ^∗^*P* < 0.05; ns, no significance.

When evaluating the diagnostic value of plasma exosomal hsa-miR-21-5p, we found that its expression was most significantly up-regulated in BC patients, but there was no significant difference in plasma-derived exosomes across lung, liver, cervical, and ovarian cancer patients (Fig. 9A). Additionally, ROC curve analysis showed that plasma exosomal hsa-miR-21-5p could distinguish healthy people from BC patients, with an area under the curve of 0.961 (95% CI [0.920–1.00], *P* < 0.05), and a sensitivity and specificity of 86.7% and 93.3%, respectively (Fig. 9B).

### Down-regulated expression of target genes TGF*β*R3 and *EGFR* in BC

**Figure 10:**
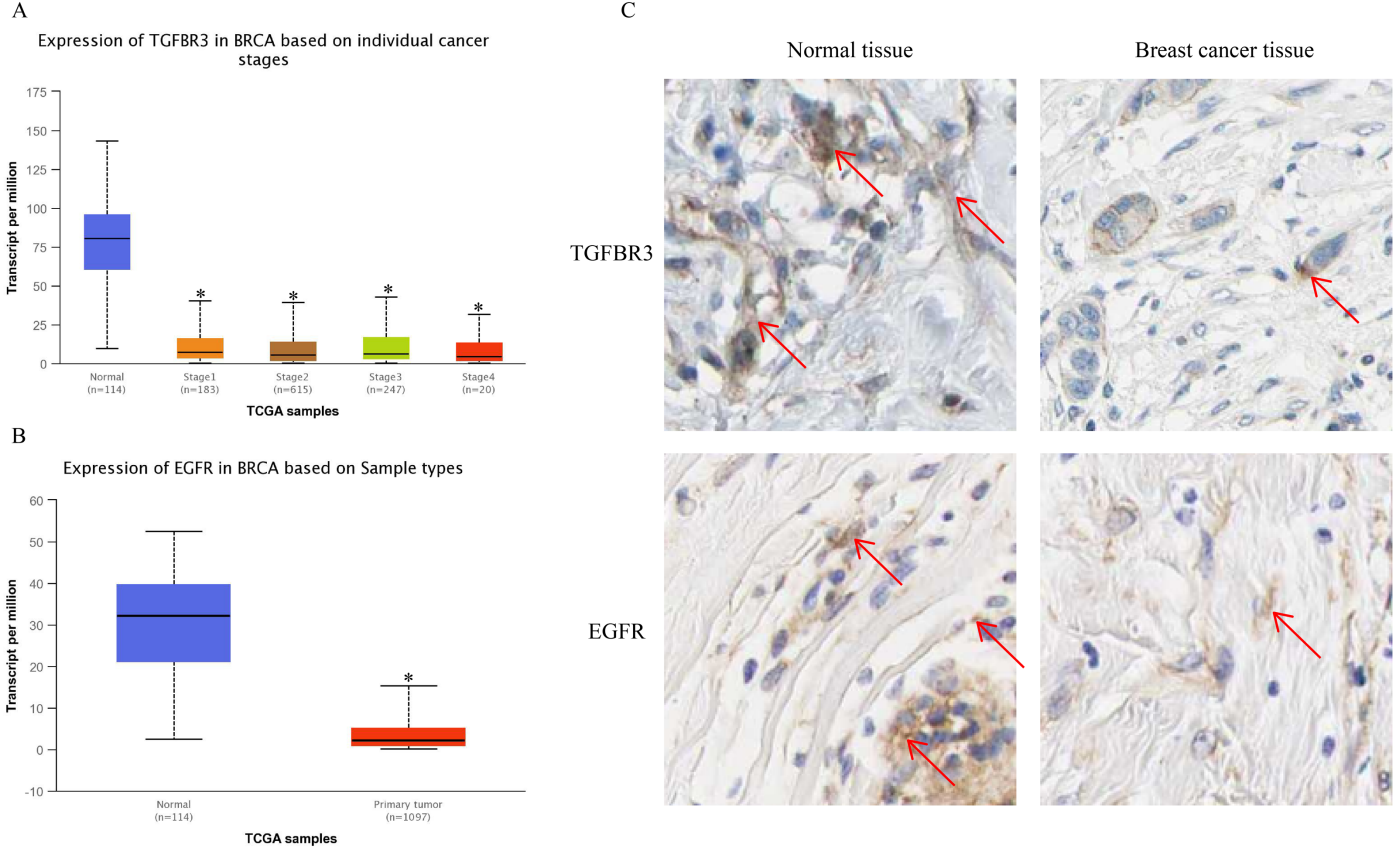
The expression of TGFβR3 and EGFR in breast cancer tissues. (A) The expression of TGFβR3 in breast cancer patients of different stages. ^∗^*P* < 0.05 *vs.* a Normal group. (B) The expression of EGFR in breast cancer tissues. (C) The expression of TGFβR3 and EGFR in the tissues of breast cancer patients.

The UALCAN analysis results showed that the expression of *TGFβR3* and *EGFR*, the potential hsa-miR-21-5p target genes, was lower in BC tissue than in normal tissue. There was no significant difference in *TGFβR3* expression across different BC stages (Figs. 10A–10B). The results of our HPA database analysis showed that at the protein level, hsa-miR-21-5p’s potential target genes (*TGFβR3* and *EGFR*) were lower in BC tissue than in normal tissue (Fig. 10C).

### *TGFβR3* is associated with overall survival (OS) in BC patients

**Figure 11:**
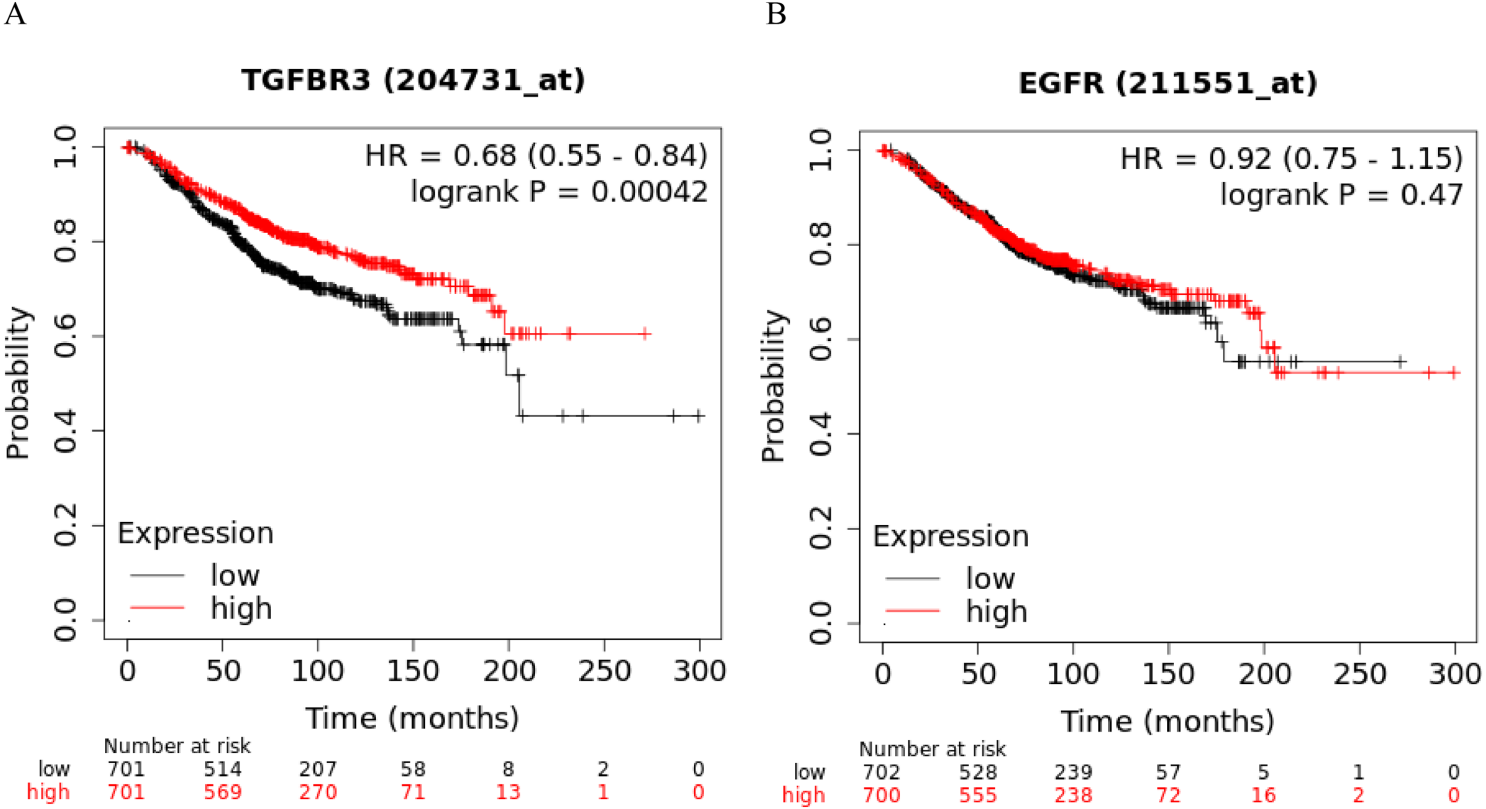
Kaplan–Meier curve analysis of potential target genes, TGFβR3 and EGFR, of hsa-miR-21-5p. (A) A Kaplan–Meier curve was used to analyze the relationship between TGFβR3 and OS in patients with breast cancer. (B) A Kaplan–Meier curve was used to analyze the relationship between EGFR and OS in patients with breast cancer.

The Kaplan-Meier curve showed that hsa-miR-21-5p’s potential target gene, *TGFβR3*, was significantly correlated with the OS of BC patients. Based on the log-rank test, we found no significant differences in the OS between *EGFR* and BC patients (Figs. 11A-11B).

## Discussion

BC is one of the most common malignant tumors in women. Its morbidity and mortality rates increase every year (Bray et al., 2018), making it the number one threat to women’s health. Therefore, BC diagnosis, treatment, and prognosis have become the focus of contemporary scholars. With recent and continuous developments and advances in gene detection, microarray and high-throughput sequencing technology play an increasingly important role in investigating biomarkers related to tumor diagnosis, treatment, and prognosis (Liu et al., 2020). Bioinformatic analysis of BC miRNA and mRNA expression profiles could quickly and effectively help us identify biomarkers for BC diagnosis.

In this study, we analyzed two datasets from the GEO database and identified 18 DE-miRNAs and 479 DE-mRNAs. After constructing the miRNA-mRNA regulatory network, we screened and selected hsa-miR-21-5p, the core network and the most up-regulated, as the subject of follow-up research. Our experimental verification results were consistent with trends in bioinformatics analysis, indicating that the hsa-miR-21-5p expression was up-regulated in tissues and plasma-derived exosomes of BC patients and in the BC cells and exosomes of cell culture media. Previous studies on miRNA-21 mainly concentrated on peripheral blood (Zhou et al., 2018) and tissue (Gao et al., 2012). The results of this study were also consistent with those of Wu, Zhu & Mo (2009). Additionally, we further verified the hsa-miR-21-5p expression in exosomes. We found that hsa-miR-21-5p expression in BC tissues was positively correlated with its expression in plasma-derived exosomes. hsa-miR-21-5p expression in plasma-derived exosomes decreased post-operatively, indicating that tumors caused the increase in plasma-derived exosomal hsa-miR-21-5p. This enriched the study of BC miRNA-21 from the perspective of plasma-derived exosomes. Moreover, our results showed that hsa-miR-21-5p’s sensitivity and specificity in BC diagnosis were 86.7% and 93.3%, respectively. Additionally, there was no significant difference in the hsa-miR-21-5p expression in plasma-derived exosomes in lung, liver, cervical, and ovarian cancer patients. This finding further confirms that plasma-derived exosomal hsa-miR-21-5p has the potential to serve as a biomarker for BC diagnosis.

In order to further explore hsa-miR-21-5p’s mechanism in BC, we used bioinformatics prediction to obtain two potential target genes: *EGFR* and *TGFβR3*. Our results show that at the protein and gene level, both *EGFR* and *TGFβR3* expression was down-regulated in BC patient tissue. We speculated that their low expression may have been caused by the negative regulation of hsa-miR-21-5p. *TGFβR3* is often used as a tumor suppressor gene for various cancers, including lung cancer (Liu et al., 2018), pancreatic cancer (Hou et al., 2021), and head and neck cancer (Fang et al., 2020). When a tumor progresses, *TGFβR3* expression decreases and is associated with a poor patient prognosis (Hou et al., 2021). *TGFβR3*, also known as betaglycan, is the largest kind of transmembrane glycoprotein distributed on the cell surface. This gene is located on human chromosome 1p33-p32 and is the most abundant receptor in transforming growth factor-β (*TGFβ*) signal transduction (Hou et al., 2021). It plays a biological role by binding to specific receptors on the cell membrane. We analyzed *TGFβR3 expression* in 1,065 BC patients in the UALCAN database. Our results showed no significant relationship between *TGFβR3* expression and the patients’ BC stage (*P* > 0.05). Dong et al.’s (2007) study of BC samples showed that the loss of *TGFβR3* expression was related to BC progression and its expression was significantly correlated with the BC stage (*P* < 0.05). Our results were different from those of Dong et al. (2007), but there are a number of potential explanations for this. For example, Dong et al. (2007) did not include the BC patients’ clinical information, such as age and race. This data could not be analyzed with the subjects included in the UALCAN database leading to inconsistent results. Our initial analysis showed that BC is a highly heterogeneous disease. Russnes et al. (2011) found clear heterogeneity across estrogen receptor-positive (ER^+^) tumors using microarray analysis at the genome, transcriptome, and epigenetic levels. Baretta, Olopade & Huo (2015) evaluated the prognostic impact of heterogeneity in hormone-receptor status in bilateral synchronous (SBC) and metachronous breast cancer (MBC) patients. In both patient cohorts they found that heterogeneity in hormone-receptor status could be used to predict OS and BC-specific It is possible that low *TGFβR3* expression may reduce the binding rate of ligand TGF-β, which survival. ER status had a greater prognostic value compared to progesterone receptor (PR) status. in turn reduces the active signal transmitted to downstream genes, leading to a decrease in the tumor suppressor effect of the TGF-β pathway and ultimately leads to a poor prognosis for BC patients.

## Conclusion

In conclusion, our study demonstrated that the plasma-derived exosome hsa-miR-21-5p could be used as a biomarker for BC diagnosis.

##  Supplemental Information

10.7717/peerj.12147/supp-1 Supplemental Information 1 18 DE-miRNAs of GSE97811

10.7717/peerj.12147/supp-2 Supplemental Information 2 479 DE-mRNAs of GSE29044

10.7717/peerj.12147/supp-3 Supplemental Information 3 52 overlapping mRNAs

10.7717/peerj.12147/supp-4 Supplemental Information 4 GO and KEGG results

10.7717/peerj.12147/supp-5 Supplemental Information 5 Code

10.7717/peerj.12147/supp-6 Supplemental Information 6 MiRNA-mRNA network

10.7717/peerj.12147/supp-7 Supplemental Information 7 RT-qPCR dataset

10.7717/peerj.12147/supp-8 Supplemental Information 8 The primers used for the real-time PCR

10.7717/peerj.12147/supp-9 Supplemental Information 9 Western blots

## Abbreviations

miRNA: microRNA
GEO: Gene Expression Omnibus
DE: differentially-expressed
DE miRNA: differentially-expressed microRNA
DE-mRNA: differentially-expressed mRNA
GO: Gene Ontology
KEGG: Kyoto Encyclopedia of Genes and Genomes
PPI: protein–protein interaction
TEM: transmission scanning electron microscope
NTA: nanoparticle tracking analysis
RT-qPCR: reverse transcription and quantitative polymerase chain reaction
ROC: receiver operating characteristic
BP: biological process
MF: molecular function
CC: cellular components
OS: overall survival time
HC: healthy control
BC: breast cancer
LIHC: liver hepatocellular carcinoma
LCa: lung cancer
CESC: cervical squamous cell carcinoma and endocervical adenocarcinoma
OV: ovarian serous cystadenocarcinoma

## Additional Information and Declarations

### Data Availability

The following information was supplied regarding data availability: The raw data are available at GEO: GSE29044 and GSE97811.
