# Supplementary figures and images for: Exosomal hsa-miR-21-5p is a biomarker for breast cancer diagnosis
Source: PeerJ. 2021 Sep 17;9:e12147. doi: 10.7717/peerj.12147 (PMC8451442; doi:10.7717/peerj.12147)

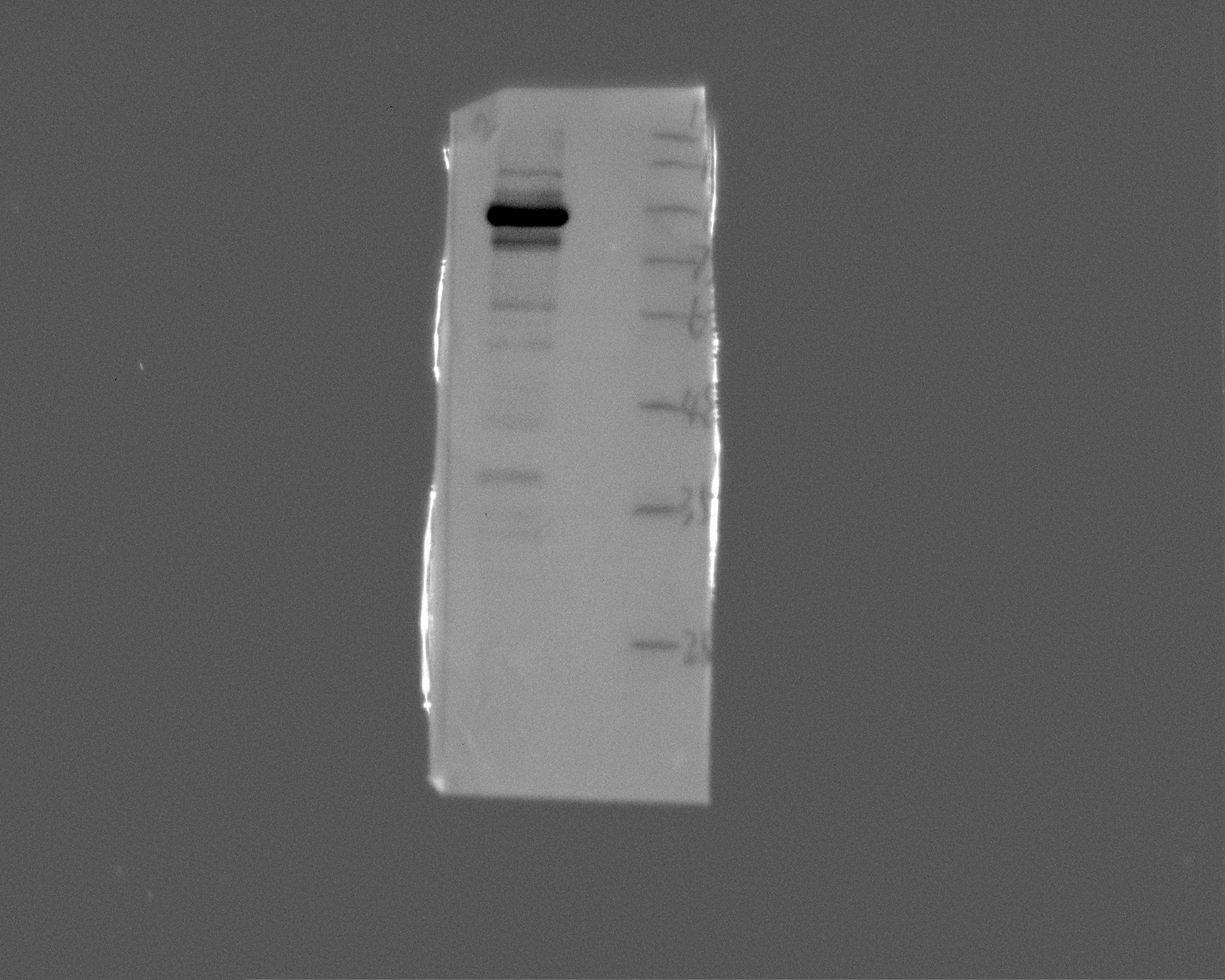

Supplement: Supplemental Information 9 [file peerj-09-12147-s009.zip › western_blotting/Calnexin.tif]

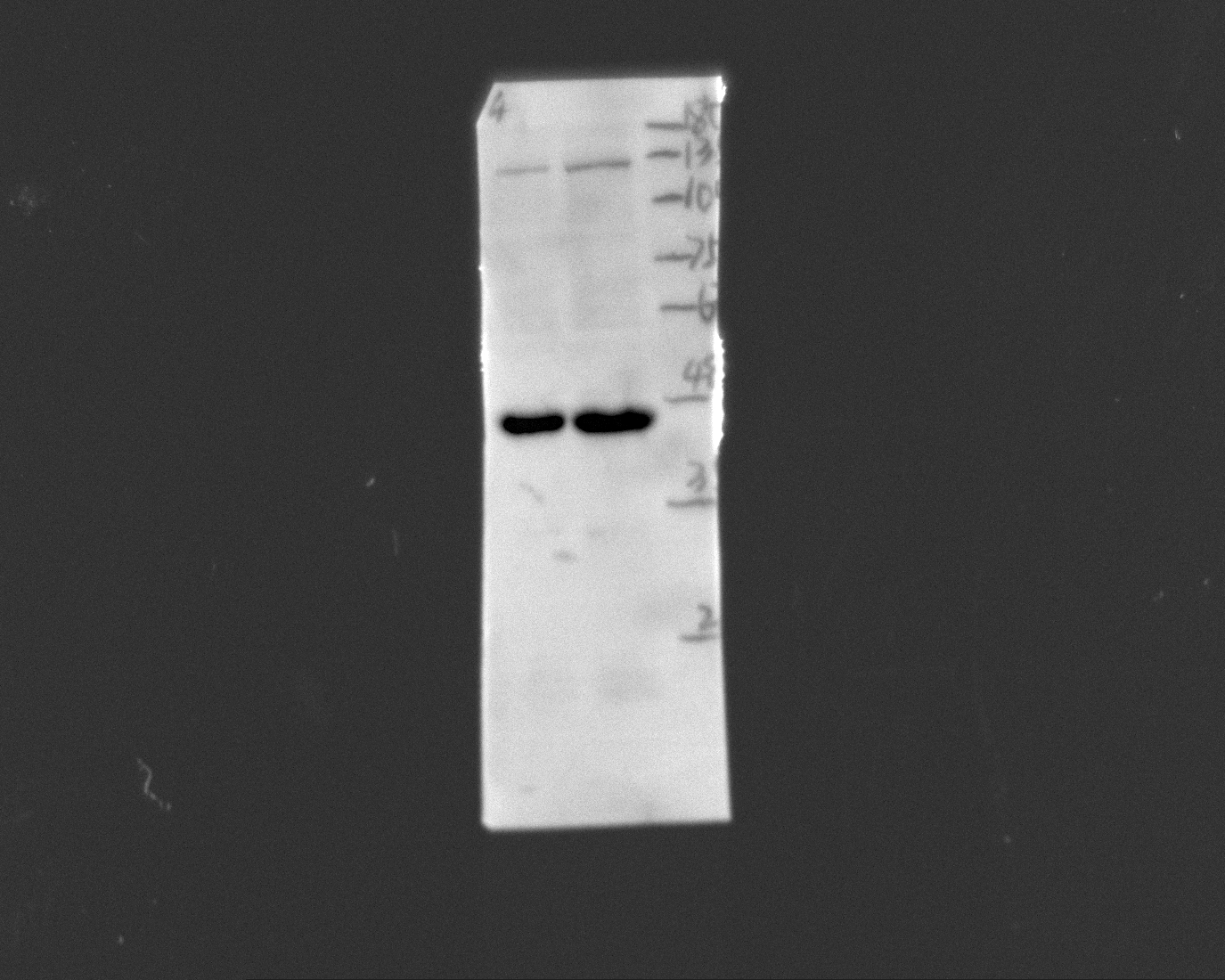

Supplement: Supplemental Information 9 [file peerj-09-12147-s009.zip › western_blotting/TSG101.tif]
